# Transcriptional Robustness Complements Nonsense-Mediated Decay in Humans

**DOI:** 10.1371/journal.pgen.1002296

**Published:** 2011-10-13

**Authors:** Claus O. Wilke

**Affiliations:** Section of Integrative Biology, Center for Computational Biology and Bioinformatics, and Institute of Cell and Molecular Biology, The University of Texas at Austin, Austin, Texas, United States of America; University of Michigan, United States of America

In eukaryotes, gene expression is a complex, multi-step process involving transcription, splicing, translation, and post-translational modifications. At each individual step, errors can occur that lead to nonfunctional and potentially toxic proteins. Therefore, eukaryotes have evolved a wide array of solutions to minimize the risk of error.

There are two fundamental strategies to minimize the production of harmful, erroneous proteins [1]: Under the first, the *global strategy*, error rates are minimized directly, e.g., through improved proofreading. Thus, the global strategy yields improved gene-expression machinery. By contrast, under the second, the *local strategy*, sequences are encoded in such a way that errors are unlikely to occur at those sites where they would be particularly harmful. Thus, the local strategy produces sequences that are robust to the deleterious effects of errors. It minimizes the deleterious effects of errors rather than error rates directly. In this issue of *PLoS Genetics*, Cusack and colleagues [2] provide an intriguing demonstration of how the local strategy can complement specific weaknesses of the global strategy.

**Figure 1:**
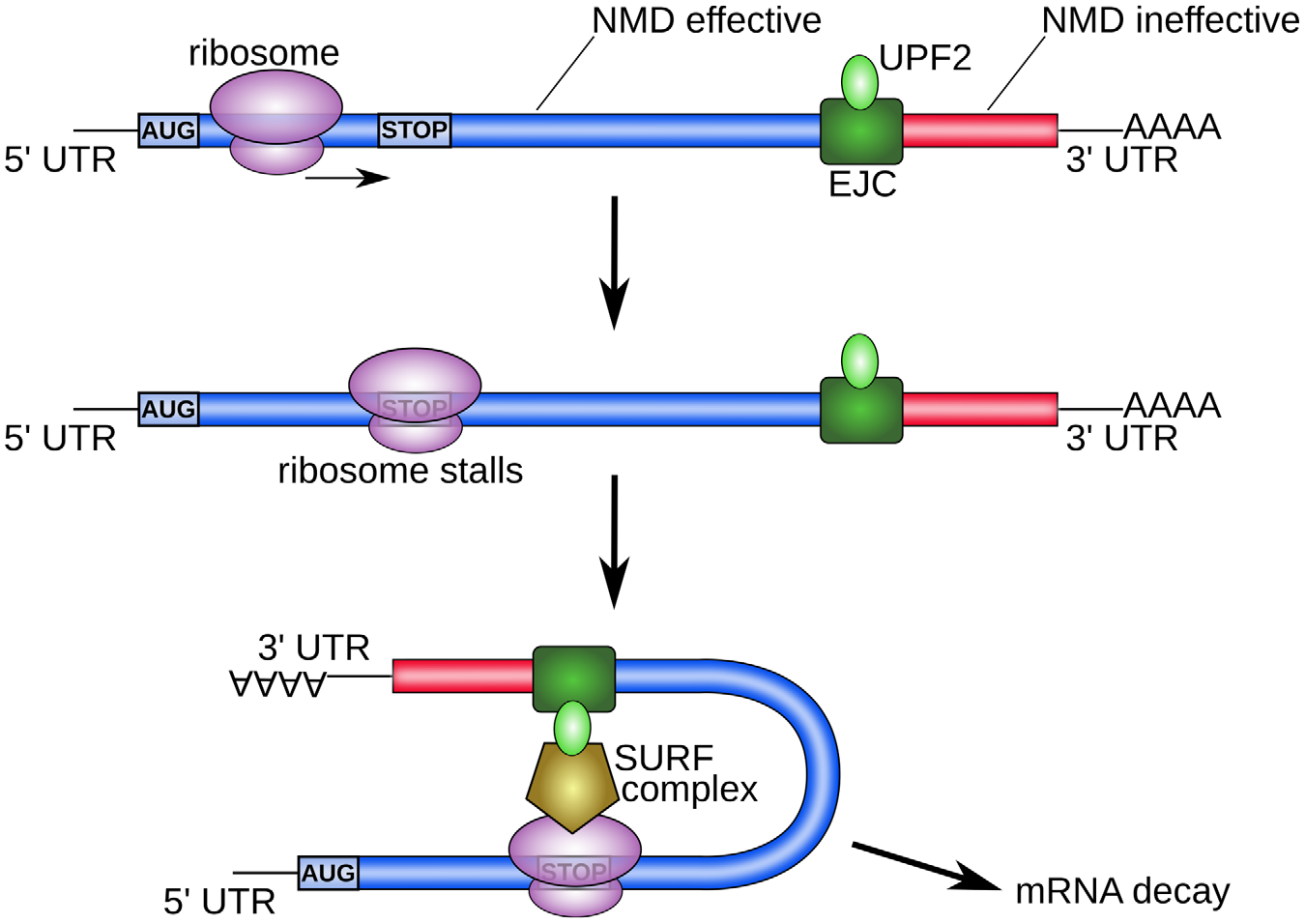
EJC-dependent nonsense-mediated decay (NMD) [4]. After splicing, exonic-junction complexes (EJCs) remain 20–24 nucleotides upstream of every exon junction. These EJCs are then bound by UPF2, one of the core proteins of NMD. When the first ribosome translates the mRNA, it displaces the EJCs. However, if the ribosome encounters a premature stop codon and stalls, it forms a complex with a downstream EJC, mediated by UPF2 and another complex called SURF. (The SURF complex is named after its constituent proteins [5].) This complex then initiates mRNA decay. Because EJC-dependent NMD requires a downstream EJC, it is only effective in the coding regions upstream of the last EJC (indicated in blue). It cannot detect any premature stop codons downstream of the last EJC (indicated in red). Note that an alternative, less potent mode of NMD takes place in the absence of the EJC [6].

Cusack et al. study how the human genome has evolved to minimize the consequences of premature stop codons introduced by transcription errors. Transcripts containing such premature stop codons are usually degraded via nonsense-mediated decay (NMD). However, the dominant mode of NMD can only detect premature stop codons upstream of the last exonic junction (EJ), because its mode of action involves exonic junction complexes (EJCs) (Figure 1). Therefore, single-exon genes and terminating exons in multi-exon genes are not well protected by NMD. Cusack et al. reason that these sequences can be protected from premature termination by encoding them in such a way that transcription errors are unlikely to introduce stop codons in the first place.

Of the 61 sense codons, 18 codons differ in exactly one nucleotide from a stop codon (see Figure 1 in [2]). Thus, these 18 codons can be converted into a stop codon by a single transcription error. Cusack et al. refer to these codons as fragile. The remaining 43 sense codons are robust. Amino acids can similarly be classified as *fragile*, *robust*, or *facultative*: amino acids that can only be encoded by fragile codons are fragile, amino acids that can only be encoded by robust codons are robust, and amino acids that can be encoded by either fragile or robust codons are facultative. There are six fragile amino acids, ten robust amino acids, and four facultative amino acids. Since the facultative amino acids can be encoded with either fragile or robust codons, one way to reduce the risk of premature termination is to encode facultative amino acids preferentially with robust codons. A second level of protection comes from amino-acid choice. Proteins can be quite tolerant to amino-acid substitutions, and thus decreasing the number of fragile amino acids in favor of either more robust amino acids or facultative amino acids encoded by robust codons will also reduce the risk of premature termination. What Cusack et al. have shown is that the frequency of fragile codons in single-exon genes is significantly reduced via both avenues when compared to multi-exon genes. Similarly, the last exons of multi-exon genes show a significant reduction in fragile codons compared to preceding exons. The effect found by Cusack et al. is in the order of 10% to 20%.

Cusack et al.'s analysis is purely statistical. In such an analysis, it is imperative to ascertain that results are not due to the influence of some confounding variable. For example, if single-exon genes differ in their GC content from multi-exon genes, for unrelated reasons, then this difference could cause an apparent reduction of fragile codons in single-exon genes. Cusack et al. have done a laudable job at ruling out a large number of such potential issues. They have also shown that comparable results are not found in the fly *Drosophila melanogaster*, for which EJC-dependent NMD is largely ineffective. As a whole, their analysis paints a convincing picture that selection removes fragile codons in sequence regions with impaired NMD.

This analysis adds to a growing body of evidence demonstrating that nature frequently chooses to minimize the deleterious effects of errors rather than the error rates themselves [3]. It remains an open question under what specific conditions selection should prefer increased robustness to errors over reduced error rates. We can speculate that evolutionary adaptations to reduce error rates will often be costly (in terms of additional energy spent) or inaccessible (if very different molecular machinery would be needed to have a substantial effect on error rates), whereas increased robustness is often free, or nearly so. In particular, in the present case, the EJC-dependent NMD would have to be replaced by a completely different mechanism to make NMD effective in single-exon genes or last exons. Such an alternative mechanism is certainly not easily evolutionarily accessible. Replacing fragile by robust codons, on the other hand, will in many cases carry no or at most a negligible selective cost.

Selection can only act to remove fragile codons if a sufficient selective benefit can be derived from replacing a single fragile codon by a robust codon. Therefore, Cusack et al.'s findings imply that erroneous premature termination of protein synthesis can generate a significant cost on organism fitness. As a consequence, we can assume that some genetic diseases in humans will be caused either entirely or at least in part by such premature terminations. However, the genes causing these diseases will be difficult to identify. They will not contain any premature stop codons, only a propensity to cause harm if a premature stop codon is accidentally introduced by the transcription machinery.
